# Micro/Mesoporous Fe_3_O_4_/Fe-Phthalocyanine Microspheres and Effects of Their Surface Morphology on the Crystallization and Properties of Poly(Arylene Ether Nitrile) Composites

**DOI:** 10.3390/ma11081356

**Published:** 2018-08-05

**Authors:** Kui Li, Dengxun Ren, Xianzhong Tang, Mingzhen Xu, Xiaobo Liu

**Affiliations:** Research Branch of Advanced Functional Materials, School of Materials and Energy, University of Electronic Science and Technology of China, Chengdu 610054, China; leekingue2014@163.com (K.L.); rendenxun2008@126.com (D.R.); txzhong@uestc.edu.cn (X.T.)

**Keywords:** micro/mesoporous, Fe_3_O_4_/FePc hybrid microspheres, adsorption and desorption, interfacial properties, poly(arylene ether nitrile) composite

## Abstract

The surface morphology of nanoparticles significantly affects the final properties and interfacial characteristics of their composites. Thus, investigations on the surface morphology of the nanoparticles is essential to fabricate improved nanoparticle-reinforced composites. Fe_3_O_4_/Fe-phthalocyanine (FePc) hybrid microspheres with micro/mesoporous structures were prepared via a solvothermal process and solvent etching method. The surface morphology and compositional distribution were respectively investigated using a scanning electron microscope (SEM) and a transmission electron microscope (TEM) to rule out that FePc monomers have been blended with Fe_3_O_4_ to form Fe_3_O_4_/FePc hybrid microspheres without serious agglomeration. The surface roughness of Fe_3_O_4_/FePc microspheres was investigated by the scanning probe microscope (SPM), and confirmed by the adsorption and desorption isotherms of N_2_. The effects of the various surface morphologies on the crystallization behavior of crystallizable poly(arylene ether nitrile) (*c*-PEN) were first employed to confirm the surface characteristics of the resulted microspheres. Results indicated that the etched Fe_3_O_4_/FePc microspheres would improve the crystallization degree of *c*-PEN, due to their much more micro/mesoporous structures than that of original Fe_3_O_4_/FePc. Then, Fe_3_O_4_/FePc hybrid microspheres reinforced PEN composite films were prepared and their interfacial compatibility was monitored using an SEM. Excellent thermal stability and improved mechanical properties were obtained by combining the etched Fe_3_O_4_/FePc and PEN matrix. The excellent surface properties and micro/mesoporous structures make the novel Fe_3_O_4_/FePc an excellent candidate of organic/inorganic hybrid fillers and micro/mesoporous materials.

## 1. Introduction

In the field of polymeric matrix composites, the interface compatibility between reinforcement and polymeric matrices is one of the most important factors that influence the composite properties [1,2,3]. When the reinforcement was an inorganic filler, especially ceramic, the interface compatibility between the reinforcement and polymeric matrix was poor [4]. Therefore, the technology of the filler modified branch has been extensively studied [5,6]. Organic/inorganic hybrids are an important method to improve the surface properties of an inorganic filler, because the organic component can increase the interface interaction between fillers and polymeric matrices [7]. Fe_3_O_4_/FePc hybrid microspheres are unique magnetic nano-fillers, and the existence of Fe-phthalocyanine (FePc) not only offered the polar organic groups, but also enhanced the dielectric property [8]. However, the size of Fe_3_O_4_/FePc hybrid microspheres by a solvothermal process is larger than 100 nm, and the relatively small surface area of microspheres makes the microsphere and polymer matrix lack compatibility at the nanometer scale.

Nowadays, humans pay more attention to micro/mesoporous structure materials for their high surface area and excellent surface properties [9,10]. The applications of micro/mesoporous structure materials are extensive in adsorption and separation, catalyst carrier, membrane separation, and energy storage applications [11,12,13,14,15]. To date, a lot of preparation methods of micro/mesoporous structure materials have been obtained including templating [16], recrystallization [17], and dealumination/desilication [18], etc. However, most of these preparation methods were post-processing with a high temperature to obtain a large number of micro/mesoporous. Moreover, the treatment may significantly destroy the organic components. Compared with these methods, solution etching is a more simple and easy way to prepare porous materials. The degree of etching can be controlled easily by tuning the etching conditions. Thus, organic/inorganic hybrid micro/mesoporous materials can be obtained with etching methods.

In this work, a novel kind of micro/mesoporous Fe_3_O_4_/FePc hybrid microspheres was designed and prepared via an etching method. The micromorphology of the etched Fe_3_O_4_/FePc hybrid microspheres were investigated in detail with SEM, TEM, SPM, and the adsorption and desorption isotherms of N_2_. To further study the effects of the micro/mesoporous morphology on the interfacial properties of their composites, a kind of crystallizable poly(arylene ether nitrile) (*c*-PEN) were selected to confirm the morphology of the Fe_3_O_4_/FePc hybrid microspheres on the crystallization. Then, the high-performance PEN was also selected to study the dielectric, mechanical, and thermal properties of Fe_3_O_4_/FePc/PEN hybrid microsphere composites.

## 2. Materials and Methods 

### 2.1. Materials

N-methylpyrrolidone (NMP, 99%) was purchased from Tianjin BODI chemicals, Tianjin, China. Tetrahydrofuran (THF, 99%), FeCl_3_·6H_2_O (99%), NaAc·3H_2_O (99%), and ethylene glycol (EG) (99.5%) were purchased from Kelong Reagent Co. Ltd., Chengdu, China. Tri-substituted-bisphthalonitrile (TPH) was prepared in our laboratory [19]. Polyethylene glycol (PEG 2000) was purchased from Tianjin Zhiyuan Reagent Co. Ltd., Tianjin, China. Two kinds of different molecular weight polyarylene ether nitriles (PEN) were synthesized in our laboratory [20,21]. 

### 2.2. Preparation of Original Fe_3_O_4_/FePc Magnetic Hybrid Microspheres

In our previous work, the synthetic procedure of Fe_3_O_4_/FePc hybrid microspheres was reported [22,23]. In this work, the synthetic route of the Fe_3_O_4_/FePc was according to following procedure with a little modification: FeCl_3_·6H_2_O (11.8 g) was dissolved in EG (200 mL) at room temperature, followed by the addition of PEG 2000 (8.15 g) and TPH (3.5 g) to form an orange solution with the help of an ultrasonic bath. The NaAc·3H_2_O (31.5 g) was slowly added into the solution with vigorous stirring for 30 min and then poured in a Teflon-lined stainless-steel autoclave. The autoclave was heated and maintained at 200 °C for 15 h, then allowed to cool to room temperature. The products were filtered and washed several times with ethanol and distilled water, then dried at 60 °C for 10 h. The original Fe_3_O_4_/FePc hybrid microsphere was obtained and labeled as *O*-Fe_3_O_4_/FePc.

### 2.3. Preparation of Etched Fe_3_O_4_/FePc Hybrid Microspheres

The Fe_3_O_4_/FePc hybrid microspheres were added into the NMP solvent treated by ultrasonic wave at room temperature and stirring for 1 h. Then, the mixture was treated at 80 °C for 24 h with vigorous stirring. After being separated, the sample was washed several times with ethanol and distilled water and dried at 60 °C for 12 h. The obtained microspheres were named as etched Fe_3_O_4_/FePc hybrid microspheres (*E*-Fe_3_O_4_/FePc). 

### 2.4. Preparation of Crystallizable PEN Coated Fe_3_O_4_/FePc Magnetic Microspheres

The crystallizable PEN (*c*-PEN) was used to obtain the coated Fe_3_O_4_/FePc microspheres [4]. First, Fe_3_O_4_/FePc hybrid microspheres (0.6 g) were added into a 100 mL three-necked round-bottom flask loaded with 18 mL THF solvent and treated using ultrasonic waves for 2 h. Meanwhile, 0.3 g of *c*-PEN was dissolved in THF with stirring. After the *c*-PEN was dissolved completely, the Fe_3_O_4_/FePc hybrid microspheres suspension was added into the *c*-PEN solution with ultrasonic waves and stirring at 70 °C for 1 h. The mixture was placed in a rotary evaporator treated with ultrasonic waves until the THF was evaporated completely at 50 °C. Finally, the sample was ground into powder in a mortar, and heated at 280 °C for 4 h. With the steps above, the *c*-PEN coated *O*-Fe_3_O_4_/FePc and *E*-Fe_3_O_4_/FePc were obtained, respectively. The obtained microspheres were labeled as *c*-PEN@*O*-Fe_3_O_4_/FePc and *c*-PEN@*E*-Fe_3_O_4_/FePc, respectively. The obtained hybrid microspheres were treated at 280 °C for 4 h for the testing of X-ray powder diffraction (XRD) and Differential scanning calorimeter (DSC). For comparison, the neat *c*-PEN was also treated under the same conditions.

### 2.5. Preparation of Fe_3_O_4_/FePc Reinforced PEN Magnetic Nanocomposites

The high-performance weight PEN (*H*-PEN) and Fe_3_O_4_/FePc hybrid microspheres were blended and the composites were obtained via a solution casting method after continuous ultrasonic dispersion. The detailed processes were as follows: a certain amount of Fe_3_O_4_/FePc hybrid microspheres were added into a certain amount of NMP and treated using ultrasonic waves for 2 h. Then, the suspension solution was slowly added into the *H*-PEN solution with vigorous stirring and with an ultrasonic wave treatment at 80 °C for 0.5 h. Subsequently, the mixture was casted on a clear glass plate and dried in an oven with a procedure of 80 °C for 1 h, 80–200 °C for 6 h, and 200 °C for 2 h and 220 °C for 1 h to remove the solvent. According to the processes, various Fe_3_O_4_/FePc reinforced *H*-PEN magnetic nanocomposite films were obtained. For simplicity, the obtained films were named as neat *H*-PEN, *O*-Fe_3_O_4_/FePc/*H*-PEN, and *E*-Fe_3_O_4_/FePc/*H*-PEN, respectively. 

### 2.6. Characterizations

The micromorphologies of the Fe_3_O_4_/FePc hybrid microspheres and PEN hybrid microsphere composite films were performed using a scanning electron microscope (SEM, JSM 5900 LV, Tokyo, Japan) and a transmission electron microscope (TEM, Hitachi H600, Tokyo, Japan). The detailed surface morphologies of *O*-Fe_3_O_4_/FePc and *E*-Fe_3_O_4_/FePc were performed using a scanning probe microscope (SPM, OLS4500, Olympus, Tokyo, Japan). The Brunauer-Emmett-Teller (BET) surface area and pore size distribution of *O*-Fe_3_O_4_/FePc and *E*-Fe_3_O_4_/FePc were explored by measuring N_2_ adsorption-desorption at 77 K on an Autosorb-1 MP Autoumated Physisorption Analyzer. 

The crystallization of the hybrid microsphere and the *H*-PEN hybrid microsphere composites were performed using X-ray powder diffraction (XRD, Rigaku (Tokyo, Japan) RINT2400 with Cu Kα radiation). The thermally induced phase transition behaviors of the microspheres and *c*-PEN composites were performed on a TA Instrument DSC-Q100 (TA Instrument, New Castle, DE, USA) with a heating rate of 10 °C/min and a nitrogen flow rate of 50 mL/min from 40 °C to 360 °C. Thermal gravimetric analysis (TGA) of the *H*-PEN-based microspheres composites was carried out using a TA Instrument TGA-Q50 (TA Instrument, New Castle, DE, USA) with a heating rate of 20 °C/min from room temperature to 800 °C under a nitrogen atmosphere. 

Dielectric property measurements of the *H*-PEN magnetic nanocomposites were carried out using a TH 2819A (Tonghui, Changzhou, China) precision LCR (Inductance, Capacitance, Resistance) meter. Furthermore, the electric breakdown strength of *H*-PEN hybrid microsphere composites was carried out using a Dielectric Withstand Voltage Tester (Air Times, Beijing, China). The mechanical property measurements of PEN microsphere composite films were investigated using a SANS CMT6104 Series Desktop Electromechanical Universal Testing Machine (SANS, Shengzhen, China). 

## 3. Results

### 3.1. The Microcosmic Morphology of the Various Fe_3_O_4_/FePc

**Scheme 1:**
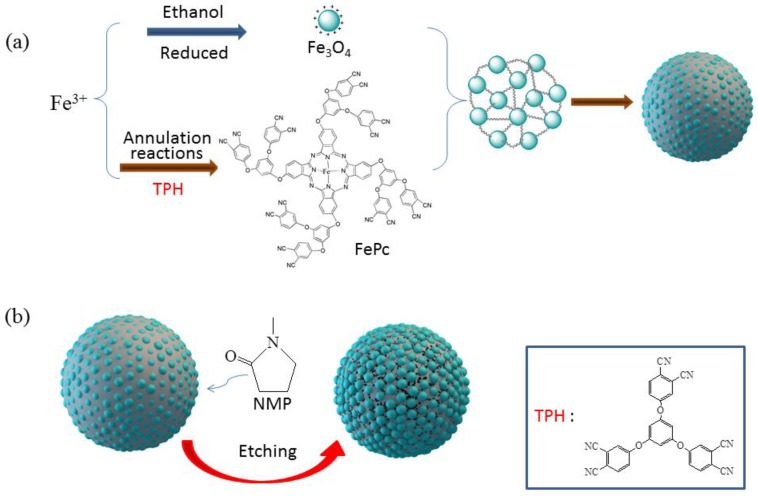
The schematic illustration of the preparation of (**a**) the original Fe_3_O_4_/FePc hybrid microspheres and (**b**) the etched Fe_3_O_4_/FePc hybrid microspheres.

**Figure 1:**
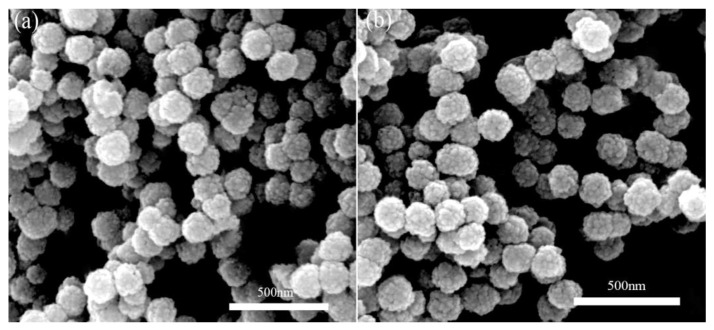
The SEM images of (**a**) *O*-Fe_3_O_4_/FePc and (**b**) *E*-Fe_3_O_4_/FePc.

The various Fe_3_O_4_/FePc microspheres were prepared using a solvothermal method, and then the microspheres were etched using NMP; the schematic illustration is shown in Scheme 1. Figure 1 shows the SEM images of *O*-Fe_3_O_4_/FePc and *E*-Fe_3_O_4_/FePc hybrid microspheres. It is obvious that the overall shape of the Fe_3_O_4_/FePc hybrid microspheres did not change too much after etching. However, the weight loss of the *E*-Fe_3_O_4_/FePc in comparison with that of *O*-Fe_3_O_4_/FePc was evaluated (≈15.4%). According to the designed Fe_3_O_4_/FePc, the weight of FePc was 51%; thus, the etched FePc was a fraction of the whole FePc in the Fe_3_O_4_/FePc microspheres. In the previous reports, the FePc was proved to be bonded with the Fe_3_O_4_ [24]. As mentioned above, it can be concluded that surplus FePc exists in the form of physical aggregation in the Fe_3_O_4_/FePc microspheres.

**Figure 2:**
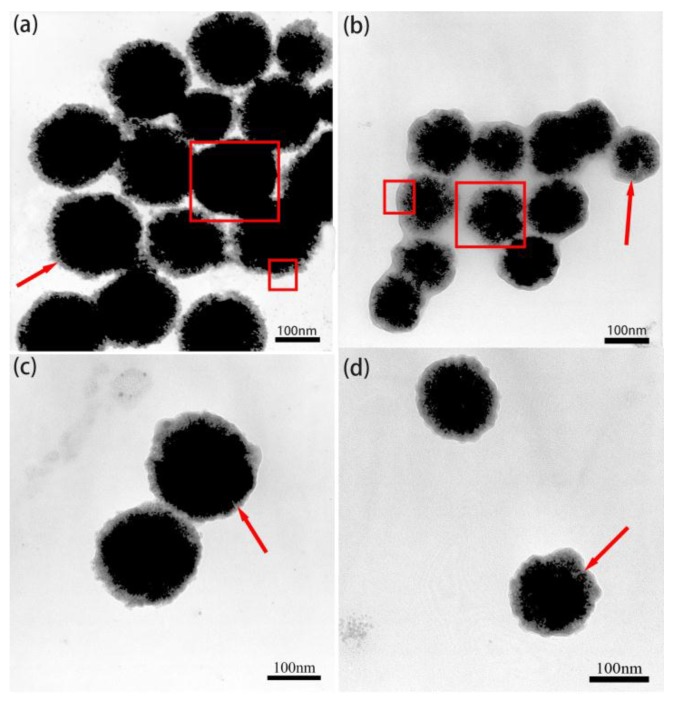
TEM images of (**a**,**c**) *O*-Fe_3_O_4_/FePc and (**b**,**d**) *E*-Fe_3_O_4_/FePc.

The microstructure of Fe_3_O_4_/FePc hybrid microspheres was investigated with TEM and is shown in Figure 2. It can be seen that Fe_3_O_4_/FePc possesses a pale edge in contrast to the dark center region. Compared with that of *O*-Fe_3_O_4_/FePc, *E*-Fe_3_O_4_/FePc shows a little smaller diameter (≈150 nm), and the thickness of the micro/mesoporous regions on the edge of microsphere increases. It indicates that the FePc embedded into the Fe_3_O_4_/FePc was etched and the frame structure of Fe_3_O_4_ remained. Meanwhile, the center of *E*-Fe_3_O_4_/FePc appeared as a pale dot (Figure 2b,d). Furthermore, from the regions marked by the red square and arrow, we can find that the surface structure of the microspheres before and after etching was obviously different. This was because hot NMP can dissolve the organic fraction of the hybrid microsphere, and so the surface of the microsphere was easily accessible by the NMP. So, after the etching, the number of micro/mesoporous regions on the surface of Fe_3_O_4_/FePc microspheres increased significantly. Due to the fact that the NMP solvent had difficulty diffusing into the center region of the microspheres, only a small amount FePc was etched and scattered mesoporous regions were observed in *E*-Fe_3_O_4_/FePc. Moreover, the fact that the microsphere morphology was observed also indicates that the organic FePc was dispersed in the hybrid microspheres, and after being treated with NMP, the fracture structures of the Fe_3_O_4_/FePc was still remaining.

Figure S1 shows the change in color of the NMP solution before and after etching, and from the photo we can see that the color of NMP solution turned into a dark transparent solution. It indicated that the organic components were dissolved in the NMP solution after 24 h of heat etching. Figure S2 shows the UV-vis spectra of the etched NMP solution after filtering. One strong sharp peak and a small peak were recorded at 350 nm and 670 nm, respectively. The former one is attributed to the TPH, the latter one is ascribed to FePc. Meanwhile, the thermogravimetric analysis of the samples before and after etching were measured and are shown in Figure S3. It is obvious that the initial decomposition temperature of *O*-Fe_3_O_4_/FePc was lower than *E*-Fe_3_O_4_/FePc. This was because the organic components of the microsphere surface are more susceptible to the thermal decomposition. What is more, the ultimate char yield at 600 °C of the *E*-Fe_3_O_4_/FePc (85.7%) is higher than *O*-Fe_3_O_4_/FePc (81.2%). This indicates that the organic components of surface of the hybrid microsphere had been etched after 24 h heat etching.

**Figure 3:**
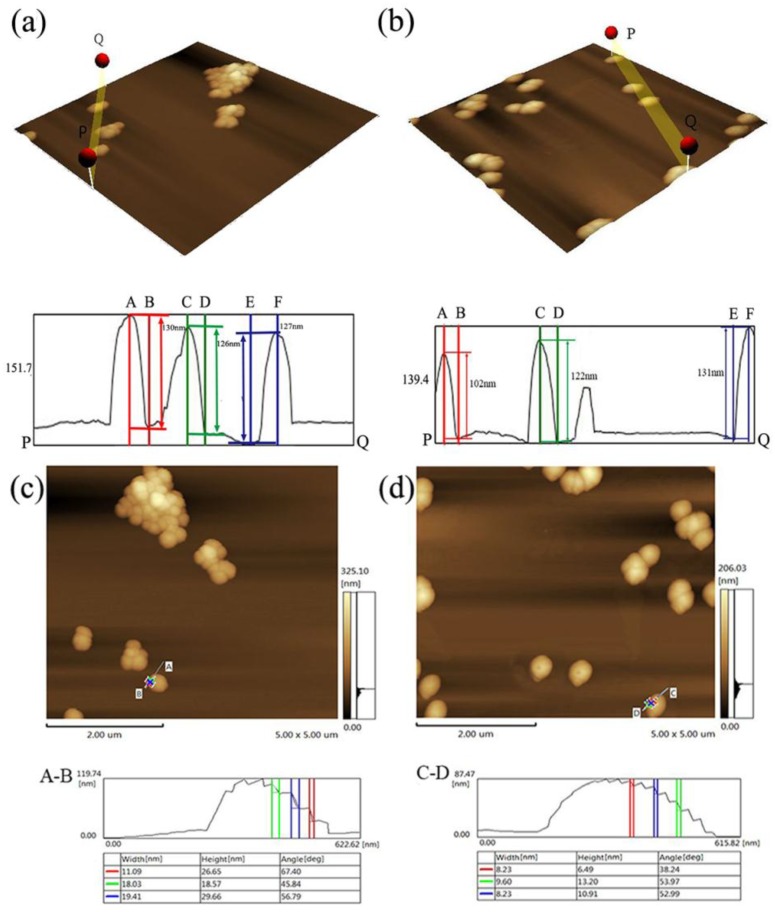
SPM 5 µm × 5 µm images of *O*-Fe_3_O_4_/FePc ((**a**) 3D and (**c**) 2D) and *E*-Fe_3_O_4_/FePc ((**b**) 3-D and (**d**) 2-D).

Further, the surface microstructure of the hybrid microspheres was studied using SPM, as presented in Figure 3. From the 3-D images (Figure 3a,b), it is obvious that the dispersion of *E*-Fe_3_O_4_/FePc microspheres was better than that of the *O*-Fe_3_O_4_/FePc. In sight of the height of each microsphere, they were all between 100 nm and 150 nm. After being etched, the size of the *E*-Fe_3_O_4_/FePc microspheres became slightly smaller than that of the *O*-Fe_3_O_4_/FePc. As is well known, the Fe_3_O_4_/FePc microspheres are composed with the inorganic Fe_3_O_4_ and organic FePc. The hot NMP solvent will significantly dissolve the organic FePc on the surface of the microspheres, which will decrease the size of the Fe_3_O_4_/FePc microspheres. The 2-D SPM images of different microspheres are shown in Figure 3c,d. It indicates that the microspheres had rough surfaces and the roughness of the *E*-Fe_3_O_4_/FePc was obviously greater than that of *O*-Fe_3_O_4_/FePc.

### 3.2. The Surface Characteristics of the Various Fe_3_O_4_/FePc

**Figure 4:**
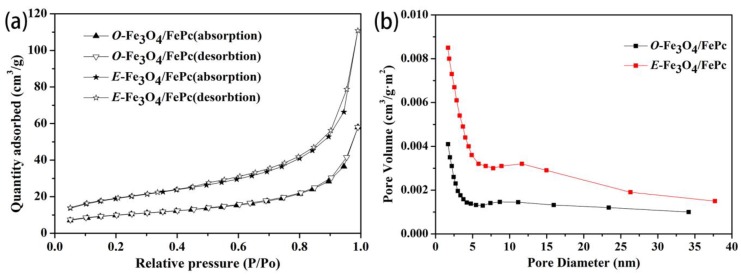
(**a**) N_2_ adsorption and desorption isotherms and (**b**) BJH isotherm curves (by adsorption) of *O*-Fe_3_O_4_/FePc and *E*-Fe_3_O_4_/FePc.

To investigate the pore structure of the various Fe_3_O_4_/FePc hybrid microspheres, nitrogen absorption and desorption experiments were carried out. N_2_ absorption and desorption isotherms of *O*-Fe_3_O_4_/FePc and *E*-Fe_3_O_4_/FePc hybrid microspheres are shown in Figure 4a; they show a small hysteresis loop. The BET surface area and single point total pore volume of *O*-Fe_3_O_4_/FePc was calculated to be 34.6 m^2^/g and 0.101 cm^3^/g. In comparison, the BET surface area and single point total pore volume of hybrid microsphere was increased to 66.7 m^2^/g and 0.171 cm^3^/g. It indicates the obvious etching effects of hot NMP solvent on the Fe_3_O_4_/FePc hybrid microspheres. The pore size distribution of Fe_3_O_4_/FePc hybrid microsphere was calculated using the Barrett-Joyner-Halenda (BJH) model (desorption), and the results are shown in Figure 4b. It shows that the small peak of two lines at about 10–11 nm, and the BJH adsorption average pore diameter (4 V/A by BET) was 11.6 nm. The difference is that the pore volume of *E*-Fe_3_O_4_/FePc between 2 nm and 20 nm was much larger than that of *O*-Fe_3_O_4_/FePc. In summary, compared to the *O*-Fe_3_O_4_/FePc hybrid microsphere, the *E*-Fe_3_O_4_/FePc hybrid microsphere presented much more pore structures, which would result in a greater surface area.

**Figure 5:**
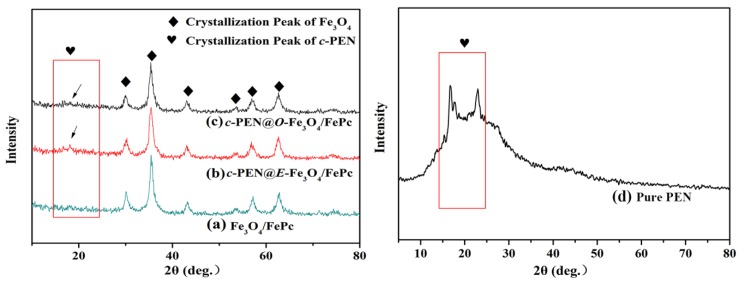
XRD curves of: (**a**) E-Fe_3_O_4_/FePc, (**b**) *c*-PEN@E-Fe_3_O_4_/FePc, and (**c**) *c*-PEN@O-Fe_3_O_4_/FePc, (**d**) pure PEN.

As reported, the nanoparticles would significantly affect the crystallization behavior of the crystallizable polymers. Especially, the interfacial interaction between the nanoparticles and the polymer matrix would directly influence the crystallization behavior. Thus, in order to further study the surface characteristics of the *O*-Fe_3_O_4_/FePc and *E*-Fe_3_O_4_/FePc hybrid microspheres, various Fe_3_O_4_/FePc microspheres with crystallizable PEN coated on the surfaces were prepared. The crystallization behaviors of the PEN on the surface of Fe_3_O_4_/FePc were investigated with XRD and DSC. The results of the XRD are shown in Figure 5. It can be observed that the main diffraction peaks of *E*-Fe_3_O_4_/FePc at 30.1° (111), 35.5° (220), 43.0° (311), 53.5° (400), 57.1° (422), and 62.7° (511) are obvious and can clearly be assigned to Fe_3_O_4_ (JCPDS card no. 19–629). For the coated Fe_3_O_4_/FePc, the characteristic diffraction peaks were all maintained and a new diffraction peak appeared at about 18°, which is attributed to the crystallization of PEN (shown in Figure 5b). In comparison, the peak at 18° for the *c*-PEN@*E*-Fe_3_O_4_/FePc is more obvious than that of *c*-PEN@*O*-Fe_3_O_4_/FePc. It indicates that the crystallization of PEN could be promoted by *E*-Fe_3_O_4_/FePc, and the promotion effects of *E*-Fe_3_O_4_/FePc were more obvious than that of *O*-Fe_3_O_4_/FePc. The obvious promotion effects of *E*-Fe_3_O_4_/FePc could be assigned to the fact that the larger surface areas offer more nucleation sites during the crystallization of *c*-PEN.

**Figure 6:**
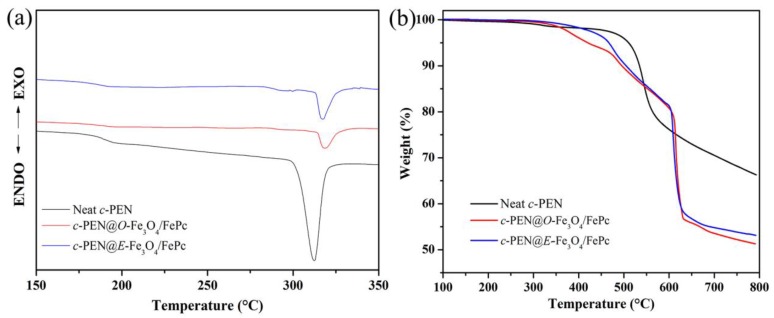
(**a**) DSC and (**b**) TGA curves of neat *c*-PEN, *c*-PEN@*E*-Fe_3_O_4_/FePc, and *c*-PEN@*O*-Fe_3_O_4_/FePc after 4 h of heat treatment at 280 °C.

DSC was also used to investigate the effects of various Fe_3_O_4_/FePc hybrid microspheres on the crystallization of PEN. The DSC curves of the neat *c*-PEN, *c*-PEN@*E*-Fe_3_O_4_/FePc, and *c*-PEN@*O*-Fe_3_O_4_/FePc hybrid microspheres are shown in Figure 6a. From the DSC curves, we can clearly see the position of the glass transition and melting transition, and the glass transition temperature (*T_g_*) of the curves are listed in Table 1. As observed, the *T_g_s* decreased with the incorporation of Fe_3_O_4_/FePc hybrid microspheres. The *T_g_* of neat *c*-PEN was 192.2 °C, and it declined to 189.7 °C with the introduction of *O*-Fe_3_O_4_/FePc. Moreover, the *T_g_* of *c*-PEN@*E*-Fe_3_O_4_/FePc declined to 188.6 °C. This can be assigned to the fact that the micro/mesoporous structure of microspheres can offer more space for PEN segment vibration, so the PEN segments would move with a lower temperature [25], and the more micro/mesoporous structure, the more obvious this phenomenon. Meanwhile, we can see clearly from DSC curves that the melting peak intensity of *c*-PEN@*E*-Fe_3_O_4_/FePc was obviously higher than that of *c*-PEN@*O*-Fe_3_O_4_/FePc. The values of melting enthalpy are also collected in Table 1, with 8.90 and 6.87 J/g for *c*-PEN@*E*-Fe_3_O_4_/FePc and *c*-PEN@*E*-Fe_3_O_4_/FePc, respectively. The melting enthalpy of neat *c*-PEN was 22.16 J/g, where the *c*-PEN accounted for one-third of the mass of *c*-PEN@Fe_3_O_4_/FePc composite, so the theoretical melting enthalpy of *c*-PEN@Fe_3_O_4_/FePc composite was 7.39 J/g, which was between the value of *c*-PEN@*E*-Fe_3_O_4_/FePc and *c*-PEN@*E*-Fe_3_O_4_/FePc. It can them be claimed that the micro/mesoporous structure of *E*-Fe_3_O_4_/FePc can improve the crystallization degree of L-PEN.

The thermal stability of the neat *c*-PEN and its microspheres was obtained using a TGA measurement, and the results are shown in Figure 6b. The initial decomposition temperature (*T*_5%_) of each curve is listed in Table 1, which were 507.7 °C, 421.2 °C, and 463.7 °C for neat PEN, *c*-PEN@*O*-Fe_3_O_4_/FePc, and *c*-PEN@*E*-Fe_3_O_4_/FePc, respectively. The initial decomposition temperature of *c*-PEN@*O*-Fe_3_O_4_/FePc was the lowest because of the decomposition of FePc and a tiny amount of PEN. Owing to the etch of FePc for *E*-Fe_3_O_4_/FePc, the initial decomposition temperature of *c*-PEN@*E*-Fe_3_O_4_/FePc increased by 10%. When the temperature was beyond 600 °C, the fast decomposition of curves can be attributed to the decomposition of Fe_3_O_4_ in the composites. In sum, due to the larger surface areas and the lower content of FePc, the *c*-PEN@*E*-Fe_3_O_4_/FePc hybrid microspheres possessed excellent thermal stability.

### 3.3. The Interfacial Characteristics and the Dielectric Properties of the Various Fe_3_O_4_/FePc/H-PEN Composites

**Figure 7:**
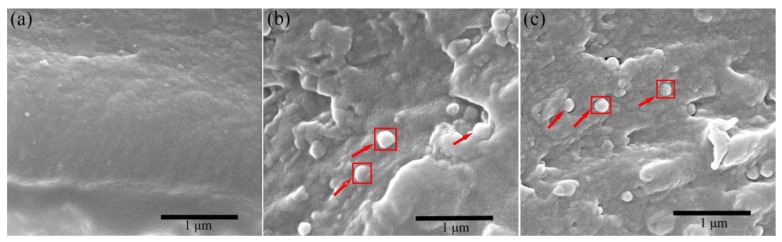
The fracture surface SEM image of (**a**) neat PEN, (**b**) *O*-Fe_3_O_4_/FePc/*H*-PEN, and (**c**) *E*-Fe_3_O_4_/FePc/*H*-PEN.

In order to study the interfacial interaction of the various Fe_3_O_4_/FePc and high-performance PEN, the various Fe_3_O_4_/FePc/*H*-PEN composites were prepared. The fracture surface of the composites was studied and is shown in Figure 7. Figure 7a–c compares the fracture surface SEM images of neat PEN, *O*-Fe_3_O_4_/FePc/*H*-PEN, and *E*-Fe_3_O_4_/FePc/*H*-PEN, which were obtained under the same conditions. It was found in Figure 7b,c that the hybrid microspheres were uniformly dispersed in the PEN matrix, and there was almost no hybrid microspheres agglomeration. Moreover, from the highlighted area with the arrow of the last two figures, we can see that there was no obvious gap between the hybrid microsphere and PEN matrix. This suggested a good interfacial compatibility between the PEN matrix and hybrid microspheres. As for the *O*-Fe_3_O_4_/FePc, the good interfacial compatibility was because of the surface organic component of *O*-Fe_3_O_4_/FePc having the similar structure and functional group with PEN chains. Different from *O*-Fe_3_O_4_/FePc, the surface organic components of *E*-Fe_3_O_4_/FePc were etched by NMP, and the good interfacial compatibility was mainly due to the large number of micro/mesoporous structures of *E*-Fe_3_O_4_/FePc. PEN chains could enter or even penetrate these micro/mesoporous regions, so that the PEN chains could intertwine with these micro/mesoporous structure microspheres. The difference is that the interfacial adhesion between the etched hybrid microsphere and PEN matrix was slightly inferior between the original hybrid microspheres and PEN matrix. This was because the pore size and distribution of micro/mesoporous regions on the surface of the *E*-Fe_3_O_4_/FePc were inhomogeneous, and in some areas, the PEN chains could not enter the channel of pores. In sum, it can be concluded that organic component of hybrid microsphere surface and micro/mesoporous structures of microspheres can improve the interfacial compatibility between microspheres and matrix.

**Figure 8:**
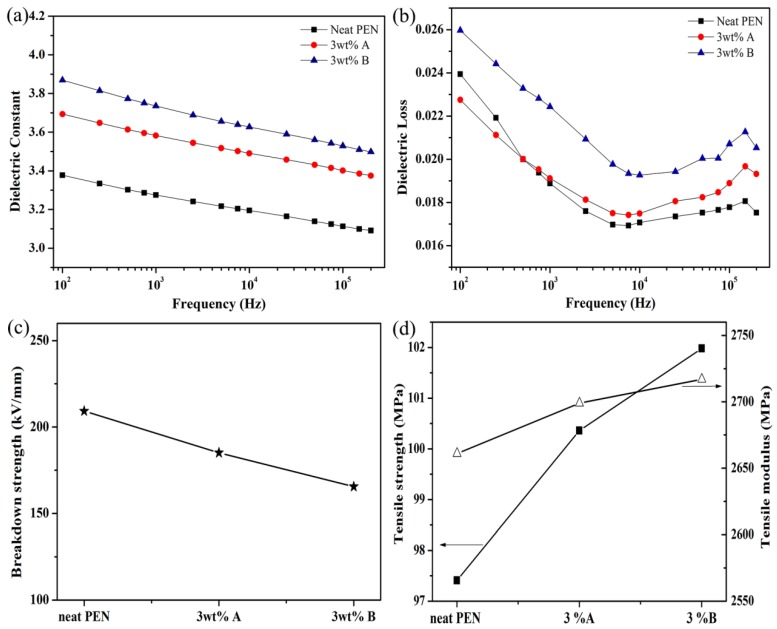
The (**a**) dielectric constant, (**b**) dielectric loss, (**c**) breakdown strength, and (**d**) mechanical property of neat *H*-PEN film, A: *O*-Fe_3_O_4_/FePc/*H*-PEN and B: *E*-Fe_3_O_4_/FePc/*H*-PEN.

The dielectric properties of *H*-PEN hybrid microsphere composite films are shown in Figure 8a–c. Figure 8a,b presents the dielectric constant and dielectric loss curves of neat PEN, *O*-Fe_3_O_4_/FePc/*H*-PEN, and *E*-Fe_3_O_4_/FePc/*H*-PEN with the change of frequency. From 100 Hz to 200 kHz, the dielectric constant of neat PEN film and PEN hybrid microsphere composite films decreased slightly with the increase in test frequency. Furthermore, with the addition of hybrid microspheres, the dielectric constant of PEN composite films increased at the same frequency. The dielectric constant of *E*-Fe_3_O_4_/FePc/*H*-PEN was higher than the value of *O*-Fe_3_O_4_/FePc/*H*-PEN. This was mainly due to the following two factors: (1) the Fe_3_O_4_/FePc hybrid microsphere possessed a high dielectric constant, and (2) the high surface area of *E*-Fe_3_O_4_/FePc increased the interface polarization between the filler and polymeric matrix. The same tendency was found for the dielectric loss of neat PEN film and PEN hybrid microsphere composite films. The dielectric loss of these films exhibited obvious frequency dependence, where a dramatic decrease was observed at low frequency, and then it increased slightly after the frequency reached 10 kHz. Nevertheless, the dielectric loss was still within a low range, with a fluctuation range of 0.015–0.026. Figure 8c shows the breakdown strength of neat PEN, *O*-Fe_3_O_4_/FePc/*H*-PEN, and *E*-Fe_3_O_4_/FePc/*H*-PEN. Compared with the neat PEN film (206 kV/mm), the addition of 3 wt% *O*-Fe_3_O_4_/FePc and *E*-Fe_3_O_4_/FePc into the PEN matrix reduced the breakdown strength, which was decreased by 9.7% and 18.6%, respectively. That was because the introduction of Fe_3_O_4_/FePc hybrid microspheres introduced some defects, which led to a reduction in the breakdown strength [26]. Furthermore, the *E*-Fe_3_O_4_/FePc, having a high surface area, possessed more micro/mesoporous regions, which introduced more defects compared with *O*-Fe_3_O_4_/FePc with the same content. 

Figure 8d shows the tensile strength and tensile modulus of neat PEN film and PEN Fe_3_O_4_/FePc hybrid microsphere composite films. As can be seen, with the addition of Fe_3_O_4_/FePc hybrid microspheres (3%), the tensile strength and tensile modulus increased slightly. The *E*-Fe_3_O_4_/FePc/*H*-PEN exhibited a higher value of tensile strength and tensile modulus compared with *O*-Fe_3_O_4_/FePc/*H*-PEN. This might be attributed to the entanglement effect of PEN molecules on the micro/mesoporous structure microsphere. Compared with traditional inorganic filler SiO_2_, the tensile strength of *E*-Fe_3_O_4_/FePc/*H*-PEN composite films (101.5 MPa with 3 wt% padding) was obviously higher than for SiO_2_/PEN composite films (92.3 MPa with 3 wt% padding) with similar content. At low filler content, the tensile strength of composite films decreased with the introduction of SiO_2_, which was different from Fe_3_O_4_/FePc/H-PEN composite films [27]. These results can also indicate the organic component of hybrid microsphere surface and micro/mesoporous structure of hybrid microsphere can improve the interfacial compatibility between microspheres and the polymeric matrix.

## 4. Conclusions

In this study, a novel micro/mesoporous structure of Fe_3_O_4_/FePc hybrid microspheres were successfully prepared using a simple solvothermal method and solution etching. SEM, TEM, and SPM images and curves of the adsorption and desorption isotherms of N_2_ confirmed that the *E*-Fe_3_O_4_/FePc presented much more micro/mesoporous structure than that of *O*-Fe_3_O_4_/FePc. The BET surface area and single point total pore volumes of *E*-Fe_3_O_4_/FePc were 66.7 m^2^/g and 0.171 cm^3^/g, respectively. The crystallization of crystallizable PEN could be improved with the introduction of *E*-Fe_3_O_4_/FePc. Moreover, the organic component of the hybrid microsphere surface and micro/mesoporous structure of hybrid microspheres can improve the interfacial compatibility between microspheres and polymeric matrix, which was confirmed with the investigation of the fracture surface morphology and mechanical properties of the composite films. Meanwhile, the incorporation of *E*-Fe_3_O_4_/FePc increased the dielectric property and mechanical property of composite films. Therefore, these excellent properties make the micro/mesoporous hybrid microspheres a good candidate in the applications of high-performance composites.

## Figures and Tables

**Table 1:** The glass transition temperature (*T_g_*), melt enthalpy (Δ*H*), and initial decomposition temperature (*T*_5%_) of neat PEN, *c*-PEN@*E*-Fe_3_O_4_/FePc, and *c*-PEN@*O*-Fe_3_O_4_/FePc after 4 h of heat treatment at 280 °C.

Samples	Neat *c*-PEN	*c*-PEN@*O*-Fe_3_O_4_/FePc	*c*-PEN@*E*-Fe_3_O_4_/FePc
*T_g_* (°C)	192.2	189.7	188.6
Δ*H* (J/g)	22.16	6.87	8.90
*T*_5%_ (°C)	507.7	421.2	463.7

## Supplementary Materials

The following are available online at http://www.mdpi.com/1996-1944/11/8/1356/s1, Figure S1: Digital photo of NMP solution (1) before etching and (2) after etching, Figure S2: The UV-vis spectra NMP solution after etching and the controlled sample of TPH and FePc in the NMP solution, Figure S3: TGA curves of the Fe_3_O_4_/FePc hybrid microspheres before and after etching.
