# Supplementary material for: Micro/Mesoporous Fe3O4/Fe-Phthalocyanine Microspheres and Effects of Their Surface Morphology on the Crystallization and Properties of Poly(Arylene Ether Nitrile) Composites
Source: Materials (Basel). 2018 Aug 5;11(8):1356. doi: 10.3390/ma11081356 (PMC6119999; doi:10.3390/ma11081356)
Supplement: Supplementary file 1 [file materials-11-01356-s001.pdf]

Supporting Information

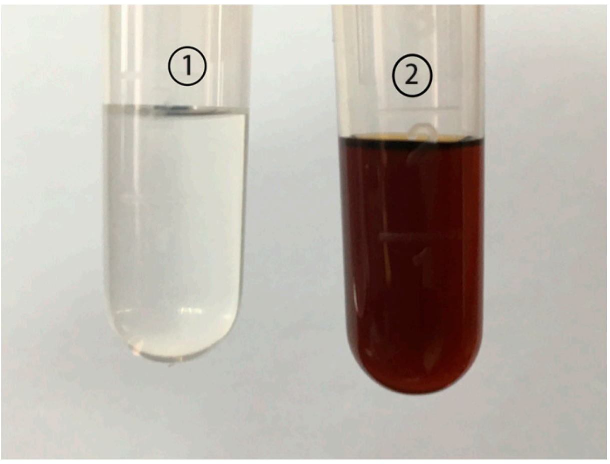

Figure S1. Digital photo of NMP solution (1) before etching and (2) after etching.

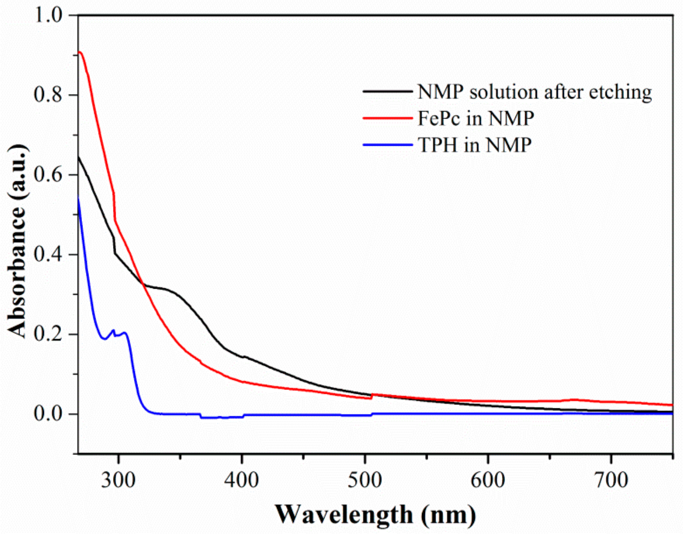

Figure S2. The UV-vis spectra NMP solution after etching and the controlled sample of TPH and FePc in the NMP solution.

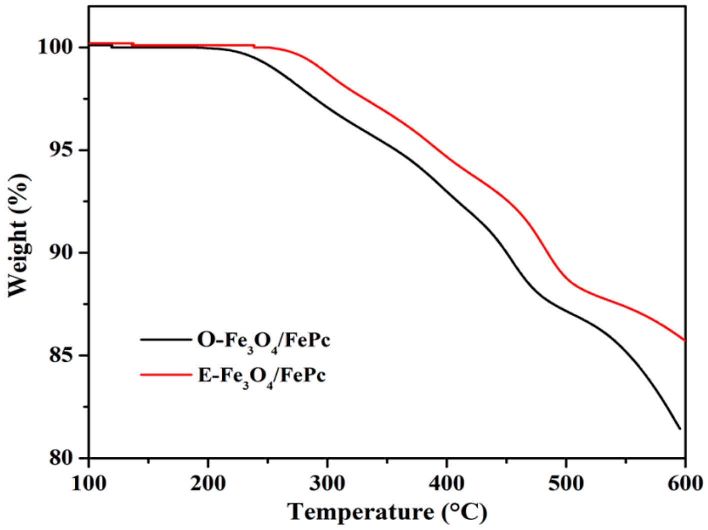

Figure S3. TGA curves of the Fe_3O_4/FePc hybrid microspheres before and after etching.
